# Immunoinformatics, molecular docking and dynamics simulation approaches unveil a multi epitope-based potent peptide vaccine candidate against avian leukosis virus

**DOI:** 10.1038/s41598-024-53048-6

**Published:** 2024-02-04

**Authors:** Siham O. Elshafei, Nuha A. Mahmoud, Yassir A. Almofti

**Affiliations:** 1 Department of Biochemistry, Faculty of Medicine and Surgery, National University, Khartoum, Sudan; 2 Department of Molecular Biology and Bioinformatics, College of Veterinary Medicine, University of Bahri, P.O. Box 1660, Khartoum, Sudan

**Keywords:** Computational biology and bioinformatics, Immunology, Microbiology

## Abstract

Lymphoid leukosis is a poultry neoplastic disease caused by avian leukosis virus (ALV) and is characterized by high morbidity and variable mortality rates in chicks. Currently, no effective treatment and vaccination is the only means to control it. This study exploited the immunoinformatics approaches to construct multi-epitope vaccine against ALV. ABCpred and IEDB servers were used to predict B and T lymphocytes epitopes from the viral proteins, respectively. Antigenicity, allergenicity and toxicity of the epitopes were assessed and used to construct the vaccine with suitable adjuvant and linkers. Secondary and tertiary structures of the vaccine were predicted, refined and validated. Structural errors, solubility, stability, immune simulation, dynamic simulation, docking and in silico cloning were also evaluated.The constructed vaccine was hydrophilic, antigenic and non-allergenic. Ramchandran plot showed most of the residues in the favored and additional allowed regions. ProsA server showed no errors in the vaccine structure. Immune simulation showed significant immunoglobulins and cytokines levels. Stability was enhanced by disulfide engineering and molecular dynamic simulation. Docking of the vaccine with chicken’s TLR7 revealed competent binding energies.The vaccine was cloned in pET-30a(+) vector and efficiently expressed in *Escherichia coli*. This study provided a potent peptide vaccine that could assist in tailoring a rapid and cost-effective vaccine that helps to combat ALV. However, experimental validation is required to assess the vaccine efficiency.

## Introduction

Avian leukosis virus (ALV) is one of the most prevalent retroviruses causing neoplasms in chickens^1,2^. ALV related to the genus *Alpharetrovirus* and subfamily *Orthoretrovirinae*^3^*.* ALVs were shown causing avian leukemia/sarcoma in birds with major economic losses in the global poultry industry^1,4^. ALV causes high morbidity rates than mortality rates In the infected flocks which negatively impacts hatchability and egg productivity^5–7^. In addition to the neoplasia, other factors contributed to mortality, including delayed maturation and diminished fertility rate, which have serious economic implications^4^.The co-infections of the virulent ALV isolates with reticuloendotheliosis virus (REV) and marek’s disease virus (MDV) are the main factor for prevalence of avian neoplastic diseases in China that hampering the future control of disease^3^. Therefore the affected flocks showed lower egg production and hemorrhages in the skin surrounding the feather follicles and phalanges^8,9^. As a result of loss of appetite, chickens characterized by weight loss, abnormal feathers, pale combs and wattles, depression, paralysis and finally death^8^. Postmortem studies revealed enlarged livers, spleens, hearts, kidneys, and congested lungs in the affected chickens^7,9,10^.

Horizontal, vertical, and genetic transmissions are the main three ways of ALV transmission^8^. The horizontal transmission occurs through direct contact with infected chickens and materials. In vertical infection, the disease is transmitted from hens to their off-springs via the eggs. Genetic transmission is through endogenous retroviruses occurred through a mendelian manner^8^. Another possible transmission route is through contaminated live and attenuated vaccines as some ALV strains were isolated from contaminated live vaccines^11^.

ALV is a member of the leukosis/sarcoma (L/S) group of avian retroviruses. It is an RNA virus family that uses the enzyme reverse transcriptase which originates the generation of the DNA provirus that is combined with the host genome during viral replication^12^. In terms of structure, ALV is a simple virus and has three chief genes located from its 5′-to-3′ end of its genome. These genes are gag/pro, pol and env genes, which code for the antigens and proteases that distinguish a different group of viron, as well as, the reverse transcriptase enzyme and the glycoprotein or the envelope protein^12^. The genomic sequence associated with an ordinance of the viral replications that flank these structural genes in the DNA provirus, forming long terminal repeats (LTRs) for the virus with promoters and enhancers^13^. Based on their viral envelope characteristics, chicken leukosis virus envelope subgroups are categorized into seven subgroups identified as A, B, C, D, E, J and K. The exogenous subgroups A, B, C, D, and J are highly pathogenic, while those of subgroup E viruses are endogenous and less pathogenic^10,14^. Virus subgroups are mainly differentiated by the receptors they use, which determine their host range and tropism. The highly pathogenic ALV-K subgroup has recently been detected in China based solely on its genomic sequence, posing a major threat to Asia's poultry industry^14,15^. In ALV genomes, subgroup differences can be primarily detected in the env gene, which binds to cell-specific receptors and dictates viral pathogenicity and tropism^16^.

Despite more than a century of ALV-related research, no vaccine and no specific treatment exist to prevent chickens from the disease. Limited anti-ALV vaccines are being developed. Among them four targeting ALV strain J, one targeting strain B, and another one targeting strain A^17^. Also, a limited protection and less immunogenicity were observed in some anti-ALV vaccine trials^17^.However defeating and preventing the ALV disease from spreading has been accomplished by eliminating affected birds, managing hatcheries and farms and implementing biosecurity measures^1,4^.

With the proliferation of big genomic and proteomic data and computing power, a successful vaccine could be developed by accessing vast information about the virus structure, protein structures, and predicted epitopes. A combination of computational methods and immunoinformatics had formed the bases for the development of multi-epitopes vaccines^18,19^. In reverse vaccinology, which analyzes a pathogen's genome and identifies its prospective antigenic proteins, multi-epitope vaccines are produced in a shorter period of time and at a lower cost, with safety and highest therapeutic efficiency and minimal adverse reactions^18,19^. In addition, to the boosting of immunogenicity, vaccines with multi-epitopes may improve immune responses because they contain epitopes representative of different target genes^20^. Moreover, this novel computational science can restrict the use of animal models and replaces some clinical trials^21^. Therefore, designing and developing multi-epitope-based peptide vaccines via computational science holds great promise for controlling and preventing the occurrence of infectious diseases in the future. Despite the advances in the field of in silico vaccines formulation, some drawbacks were emerged. For instance, the in silico approaches cannot predict lipids or polysaccharides as they are significant active constituents in proteins^22^. Also predictions of optimum antigen-processing sites are not well addressed and clarified by many epitope prediction tools^23^. On the other hand, common traditional vaccines might use inactivated or attenuated forms of microorganism, which might provide adverse effects and autoimmune diseases in infected hosts. Also the development of traditional vaccines candidate such as chimeric virus vaccines, live attenuated vaccines, virus-like particles (VLPs), subunit vaccines and nucleic acid vaccines is complex process, expensive, time consuming, need complicated in vitro and in vivo studies for evaluating their potential efficacy^24,25^.

Hence, this research targeted the use of immunoinformatics tools to predict multi epitopes vaccine that could efficiently combat the ALV infection. The proteome of ALV was investigated, evaluated and analyzed for epitopes prediction. The results demonstrated that the constructed vaccine has successfully comprised all the most favorable factors to be a potential vaccine candidate, which further requires experimental validation. This study provided good theoretical basis essential for developing a new vaccine strategy and extending the current knowledge in controlling ALV.

## Materials and methods

**Figure 1:**
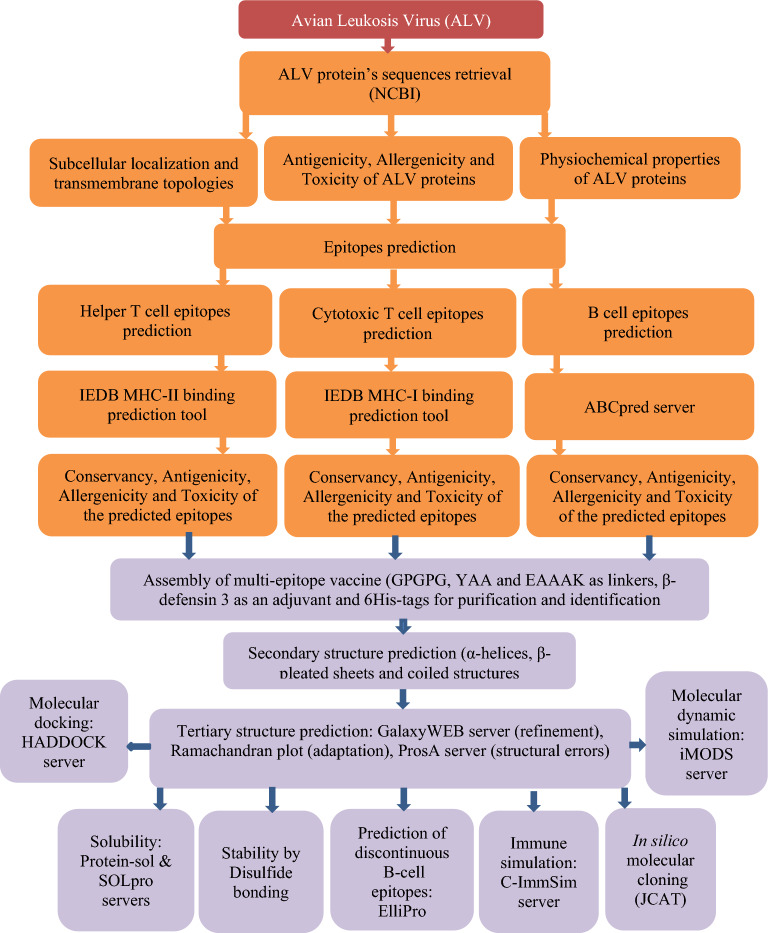
Schematic flowchart providing the overall steps used for designing the ALV multi-epitope based peptide vaccine.

The immunoinformatics steps for the in silico vaccine design were visualized in the flow chart presented in Fig. 1.

### ALV protein’s sequences retrieval

The ALV demonstrated three proteins: polymerase protein, envelope protein, and transacting factor protein with the following accession numbers NP_040550.1, NP_040548.1, and NP_040549.1, respectively. The sequences of these three proteins were retrieved from the National Center for Biotechnology Information (NCBI) at (https://www.ncbi.nlm.nih.gov/protein)^26^.

### Physiochemical properties, antigenicity, allergenicity, and toxicity of the retrieved proteins

A physiochemical profile of each retrieved protein was assessed using the Protparam server (http://web.expasy.org/protparam/)^27^. The potent antigenicity was determined using the VaxiJen v2.0 server (http://www.ddg-pharmfac.net/vaxijen/) based on adefault viral threshold of 0.4 for each antigenic protein^28,29^. Allergenicity and toxicity of the proteins were assessed using the AllerTOP server (http://www.ddg-pharmfac.net/AllerTop/)^30^ and the ToxinPred server (http://crdd.osdd.net/raghava/toxinpred/)^31^, respectively. The same servers were later used to assess the physiochemical properties, antigenicity, allergenicity and toxicity properties of the constructed vaccine.

### Subcellular localization and transmembrane topologies of virus proteins

Subcellular localization of viral proteins is considered as an important clue to the function of the immune cells and judging the potential efficacy of vaccine targets^32^. In addition, surface-localized proteins are among the best candidates for the recombinant vaccine, since they are the first molecular patterns of pathogens that contacted by the host immune system^32^. For the detection of the viral protein subcellular localization, the Phobius server (https://phobius.sbc.su.se/index.html) was used^33^. The server provided a combination of a transmembrane topology (TMHs) and a signal peptide predictor.

### Epitopes prediction and conservancy

A total of 50, 13, and 3 strains sequences were retrieved for the polymerase, envelope, and transacting factor protein, respectively. These strains were used for epitopes conservancy and were presented in Table 1. BioEdit program version 7.2.5, is a multiple sequence alignment (MSA) tool, was used to align each protein strains sequences^34^. The analysis of the aligned sequences was conducted in order to identify the conserved epitopes that effectively act against B and T lymphocytes. Epitopes that had 100% conservancy (no mutations) among the strains were selected for further analysis, while non-conserved epitopes were excluded.

**Table 1:** The total number of the retrieved strains of the polymerase, envelope, and transacting protein of ALV with their accession numbers.

Polymerase protein	Polymerase protein	Polymerase protein	Polymerase protein	Envelope protein	Transacting protein
*NP_040550.1	AKP18447.1	ADO34849.1	BAC78443.1	*NP_040548.1	*NP_040549.1
AKP18453.1	BAM15580.1	ADO34846.1	QEJ82123.1	AAA91268.1	AAA91267.1
AKP18448.1	BAM68487.1	ADO34843.1	QEG03935.1	AIY32623.1	Q04221.1
AIY32622.1	BAM68485.1	AHI49963.1	AWO14323.1	BAM68473.1	
ADP21278.1	BAM68481.1	AFG30999.1	ARH12433.1	AYN55358.1	
ADP21275.1	BAM68477.1	ASM94215.1	ARH12430.1	ABX39002.1	
BAK64409.1	BAM68472.1	AMP18914.1	AAA91269.1	ABX38999.1	
AYN55373.1	BAM68470.1	AEF97638.1	ABX39004.1	ABX38996.1	
AYN55371.1	BAL70360.1	AHC94804.1	ABX39001.1	ADN85628.1	
AYN55368.1	BAL70357.1	AIM55740.1	ABX38998.1	ADO34853.1	
AYN55365.1	BAL70354.1	BAA22090.1	ABC94886.1	ABC94884.1	
AYN55359.1	BAL70351.1	BAL70337.1		AYC62336.1	
ANW72069.1	AIL52058.1	BAK64246.1		WGM49020.1	

*Reference sequence.

### B cell epitopes prediction

To identify the B cell epitopes from the three ALV proteins, the ABCpred server (https://webs.iiitd.edu.in/raghava/abcpred/ABC_submission.html) was utilized^35,36^. The reference sequence of each protein was used as an input into ABCpred server. The default thresholds (0.51), as well as 12mers epitope length were used for the prediction strategy. VaxiJen v2.0, AllerTOP, and ToxinPred servers were further used to assess the antigenic, allergenic and toxic potentiality of the conserved B cell epitopes, respectively.

### T cell epitopes prediction

Based on Immune Epitope Database (IEDB) analysis resources at (https://www.iedb.org/), different T cell epitope prediction tools were analyzed^37,38^. The reference sequence was used as an input for each protein analysis. The data for epitopes that interacted with the major histo-compatibility complex class I and II (MHC-I and MHC-II) is not yet organized in the IEDB resources for chicken alleles. Accordingly, the human alleles were exploited to predict epitopes from the ALV-retrieved proteins interacting with T cell epitope as previously described^39,40^.

#### Cytotoxic T cell epitopes prediction

The IEDB prediction method at (http://tools.iedb.org/mhci/) provided a number of MHC-I binding prediction methods. In this study, the prediction of the MHC-I interacted alleles were obtained by Artificial Neural Network, NetMHC (ANN)^41^. The human reference alleles sets (HLA-A, HLA-B, and HLA-C) were used for the prediction process. Conserved epitopes with a score equal to or less than 1 (≤ 1) percentile rank with nine amino acids length bound to alleles were only analyzed. The conserved cytotoxic T-cell epitopes were further assessed for antigenicity, allergenicity and toxicity predictions.

#### Helper T cell epitopes prediction

The IEDB MHC-II binding prediction tool (http://tools.iedb.org/mhcii/) was used to investigate the reference sequence of ALV proteins for epitope prediction against MHC-II^37,38^. The human alleles reference sets (HLA-DP, HLA-DQ, and HLA-DR) were employed to search for promising epitopes. The analysis comprises the Neural Networks Align method, NetMHCII, version 2.2 (NN-align)^37,38,41^. The approach was used to find potential epitopes having a percentile rank score equal to or less than 10 (≤ 10). The core sequence and peptide lengths were set to 9 and 18 amino acids, respectively. The antigenic, allergenic, and toxic evaluation of the conserved helper T cell epitopes was carried out using VaxiJen v2.0, AllerTOP, and ToxinPred servers, respectively.

### Assembly of the multi-epitope vaccine

The primary assembly of the vaccine sequence was accomplished fusing the B and T cells predicted epitopes that demonstrate conservancy, antigenicity score more than 1 and were shown to be non-allergenic and non-toxic. The elected B and T helper epitopes were fused by the GPGPG linkers while the T cytotoxic epitopes were fused by the YAA linkers^42^. The 5′-amino terminal of the vaccine was supported by the β-defensin 3 (Q5U7J2) as an adjuvant after separation with the EAAAK linker. Moreover, the sequence was provided with the 6His-tag for purification and identification of the vaccine upon expression^42–45^.

### Secondary and tertiary structures prediction of the vaccine

Predicting the secondary structure of the vaccine is available for free on the raptor X server (http://raptorx.uchicago.edu/)^46^. The secondary structure (SS), disorder regions (DISO), and solvent accessibility (ACC) played crucial roles in predicting α-helices, β-pleated sheets, and coiled structures, respectively. For the tertiary structure prediction, the vaccine sequence was submitted to the same raptor X server^46^. The obtained results were received in the form of a PDB file that was further used for vaccine sequence refinement and adaptation.

### Vaccine tertiary structure refinement and adaptation

Refinement of the vaccine's tertiary structure was carried out using the GalaxyWEB server (http://galaxy.seoklab.org/)^47,48^. The structured model is made more dynamic and effective by utilizing the side chains, establishing them and repacking these side chains to achieve relaxation of the structured model^47,49^. As a result, the refinement and the structure's physical quality were improved. In the SAVES server, the Ramachandran plot (https://saves.mbi.ucla.edu/results?job=869538&p=procheck) was used to analyze the model validation^50^, and the ProsA server (https://prosa.services.came.sbg.ac.at/prosa.php) was used to analyze the errors in the protein structure^51,52^.

### Determination of the solubility of the vaccine

Protein-sol server (https://protein-sol.manchester.ac.uk/) consists of a set of predictive algorithms and a theoretical computation was used to measure the solubility of the vaccine protein^53^. The vaccine solubility was assessed in comparison with *Escherichia coli* proteins, which have an estimated solubility of 0.45. Thus, proteins with solubility score greater than 0.45 are therefore considered soluble^53^. To confirm the solubility of the vaccine, the SOLpro server (http://scratch.proteomics.ics.uci.edu/) was further employed^54^. For a protein to be soluble in the SOLpro server the probability should be ≥ 0.5 and insoluble protein scores < 0.5.

### Determination of the stability of the vaccine

The disulfide bonding in a given protein between the cysteine residues plays an important role in strengthening of the protein’s geometric conformation and enhances its extensive stability^55^. Disulfide-by-Design 2.0 (DbD2) (http://cptweb.cpt.wayne.edu/DbD2/) is a web-based tool that facilitates designing disulfide bonds in vaccine construct by substituting particular amino acid with cysteine in high-mobility and unstable regions of proteins^55^. This was followed by formation of disulfide bonds between cysteine residues. The parameters such as the intra-chain, inter-chain and build C_β_ for Gly were chosen. The angle (− 87° or + 97° ±) was set to 30 and C_α_–C_β_–S_γ_ angles (114.6° ±) was set to 10 for proper prediction of the bonds^55^.

### Immune simulation

For mimicry of the immune response and immunogenicity of the ALV vaccine in the host, C-ImmSim server (https://kraken.iac.rm.cnr.it/C-IMMSIM/) was used^56^. Two injections were given with time step set at 1 and 90 (the server provided each time step as 8 h while the time step 1 represents the injection at time = zero). The other simulation parameters were set to default. The measure of diversity (Simpson index, D) was interpreted from the plot^56^.

### Molecular dynamic simulation (MD)

iMODS server (https://chaconlab.org/multiscale-simulations/imod) was used to analyze the collective motions of protein vaccine^57,58^. A normal modes analysis (NMA) in internal coordinates is conducted by the server to determine the stability of the vaccine protein. This server structured the dynamics of the protein complex and provided various results data, such as deformability, eigenvalues, B-factors, variance maps, co-variances, elastic networks in the atoms, and residue indexes in terms of magnitude and direction^57,58^.

### Prediction of discontinuous B-cell epitopes

The ElliPro in the IEDB (http://tools.iedb.org/ellipro/) was used to predict the discontinuous B cell epitopes^59^. ElliPro tool predicts discontinuous and linear antibody epitopes based on the protein 3D structure. The prediction method was based on the default parameters of the sever^59^. For instance, the minimum score and the maximum distance (Angstrom) of the selected epitopes prediction were set to 0.5 and 6, respectively.

### Active sites detection in the vaccine structure

Searching for a ligand-binding region on a protein is an essential step prior to molecular docking process. The process primarily based on multiple factors such as detection of hydrophobic or hydrophilic interactions, salt bridges and electrostatic and hydrogen bonding interactions. The computed atlas of surface topography of proteins (CASTp 3.0) website (http://sts.bioe.uic.edu/castp/index.html?3igg) was exploited to determine the vaccine’s active regions^60,61^. The default probe radius of 1.4 ˚A was used.

### Molecular docking of the vaccine protein with chicken TLR7

Protein–protein interaction is essential for functioning of many biological molecules^62^. Analyzing the complex structures formed between these molecules is of great importance to assess the molecular interactions or the affinities between these molecules. Toll-like receptors (TLRs) are considered as recognition receptors that play a paramount role in recognition of pathogen. In birds, there are ten genes encoding for TLRs, among them, TLR7 was chosen for the docking with the vaccine construct since it is a viral-sensing TLR^63^. Thus the designed vaccine was docked against the chicken TLR7 using the HADDOCK 2.4 server (https://www.bonvinlab.org/software/haddock2.4/)^62^. Refinement interface in HADDOCK server was used to provide the accurate cluster. PRODIGY web server (https://wenmr.science.uu.nl/prodigy/)^64,65^ was used to calculate the binding affinities of the best chosen clusters at 25 °C. Finally, the interaction between the vaccine and the chicken TLR7 was visualized by PDBsum server (https://www.ebi.ac.uk/thorntonsrv/databases/pdbsum/Generate.html)^66^.

### In silico molecular cloning and codon adaptation

The in silico cloning ensures that a particular host would express the vaccine protein upon cloning in suitable vector^67^. To facilitate successful cloning, optimization process and cloning of the vaccine construct in the expression vector were performed. The optimization comprises the elimination of different restriction enzymes cleavage sites, prokaryotic ribosomal binding sites, and rho-independent terminators of transcription in the sequence of the vaccine^67^. A reverse translation of the vaccine protein sequence into a DNA sequence was performed with the Java Codon Adaptation Tool (JCAT) (http://www.prodoric.de/JCat) because cloning uses DNA rather than proteins^67^. The codon adaptation index and the GC content were in ranges of 0.8–1.0 and 30–70%, respectively. The sequences of the restriction enzymes Xho1 (5-CTCGAG-3) and BamHI (5-GGATCC-3) were added at the 5’and the 3’ ends of the DNA, respectively. A restriction cloning module from SnapGene (https://www.snapgene.com/)^67^ was used to clone the DNA sequence located between the restriction sites of BamHI and Xho1 in the pET-30a(+) vector.

## Results

### Characteristics of the virus proteome

Polymerase, envelope, and transacting factor proteins from the ALV were retrieved from the NCBI database. These three proteins were found to be stable and hydrophilic using the ProtParam server. The VaxiJen server was used to determine and prove their antigenicity. The three proteins were used as inputs to predict B and T cell epitopes for designing the vaccine against ALV. All the physical and chemical features of the three proteins were provided in Table 2.

**Table 2:** Physical and chemical properties, antigenicity, and number of the predicted transmembrane helices of ALV proteins.

Viral protein	Accession number	Molecular weight (Kilodalton)	Instability index#	Aliphatic index	Theoretical pI	No amino acids	Extinction Coefficient	GRAVY†	Vaxijen antigenicity$	Subcellular localization and TMHs
Polymerase partial	NP_040550.1	98.630	38.85	92.01	8.17	895	167,410	− 0.157	0.5453	NC with no TMH
Envelope protein	NP_040548.1	66.444	41.90	88.96	8.09	606	92,370	− 0.104	0.4782	C with 1TMH
transacting factor @	NP_040549.1	14.098	72.84	76.91	12.05	123	38,500	− 0.754	0.4504	NC with no TMH

*THMs* Transmembrane helices, *NC* not cytoplasmic, *C* cytoplasmic.

@The protein contains no Phe, Tyr, and Asp. #instability index < 40 considered the protein stable; **†**GRAVY negative sign indicated the protein is hydrophilic; **$**the threshold for the Vaxijen antigenicity is 0.4 and the three proteins were shown to be non-allergic. THMs: Transmembrane helices. NC: not cytoplasmic. C: cytoplasmic.

### Multiple sequence alignment and epitopes conservancy

The ClustalW program provided in the Bioedit tool was used for multiple sequence alignment (MSA) of all retrieved strains. MSA was exploited to search for conserved epitopes among the retrieved stains from polymerase, envelope, and transacting factor proteins. Epitopes length that was not broken by mutated amino acids from other strains is considered conserved epitope. During the MSA, the retrieved strain sequences demonstrated high epitopes conservancy.

### Linear B-cell epitopes prediction

The ABCpred server received the reference sequences from each protein. In the server, a trained recurrent neural network provided the predicted B-cell epitopes based on their scores. Generally, an epitope passing the threshold of 0.51 is more likely to have a higher peptide score. Based on the ABCpred server, 39, 29, and 10 epitopes were predicted from the polymerase, envelope, and transacting factor proteins, respectively. After assessing the antigenicity, allergenicity and the toxicity of the predicted epitopes from each protein, 11, 10 and 6 epitopes from polymerase, envelope and transacting proteins were chosen as B cell epitopes, respectively. These epitopes were provided in Table 3.

**Table 3:** The predicted B cell epitopes and their antigenicity scores.

Protein	Sequence	Start	Score*	Vaxijen antigenicity#
Polymerase protein	VEKELQLGHIEP	38	0.63	1.9908
	VNAKLVPFGAVQ	77	0.51	1.746
	WNLDMKMAWREI	291	0.73	1.5822
	AGVNPRGLGPLQ	620	0.62	1.5003
	IARPLHVSLKVR	425	0.74	1.381
	IWQTDFTLEPRM	632	0.66	1.2853
	TLEPRMAPRSWL	638	0.59	1.2682
	PLMVLDLKDCFF	102	0.78	1.2436
	LDLKDCFFSIPL	106	0.7	1.2332
	VLEPLRLKHPSL	164	0.84	1.1143
	KACNISMQQARE	593	0.8	1.1041
Envelope protein	VGFRPQGVPWYL	193	0.72	1.7176
	ACWSVKQANLTT	445	0.83	1.6310
	LTGYPGKTSKKD	9	0.84	1.4026
	KTPLLPTRVNYI	35	0.65	1.3434
	ISGITGGCVGFR	185	0.64	1.3339
	YNCSQVGRQYRC	256	0.66	1.3537
	LWITWANRTGQT	73	0.6	1.3105
	TGIRRKRSTSHL	395	0.68	1.1582
	LPTRVNYILIIG	39	0.6	1.0656
	PGKTSKKDSKEK	13	0.61	1.0458
Transacting protein	LLMWGVVCILPW	78	0.82	0.9497
	RNQQRSGESTKG	19	0.8	0.9436
	LPQVRSASRNQQ	11	0.78	0.8661
	GVVCILPWRGWE	82	0.73	0.8582
	PQPRPLLLLMWG	71	0.67	0.7729
	KPLAHPAISAEQ	53	0.62	0.4698

*The default score of the ABCpred server was 0.51 and the length of the predicted epitopes was12mers. ^#^The Vaxijen server antigenicity threshold was 0.4. All the tested epitopes were nonallergeic and nontoxic.

### Cytotoxic T lymphocyte epitopes prediction

Based on the reference sequences of polymerase, envelope, and transacting factor, multiple epitopes were predicted against human alleles (HLA-A, HLA -B, HLA-C) using IEDB MHC-1 binding prediction tools. Antigenic, allergenic, and toxic effects were then assessed for the predicted epitopes. A total of 6, 11, and 15 epitopes were obtained from the polymerase, envelope, and transacting factor proteins, respectively, and were elected as T cytotoxic cell epitopes due to their high antigenicity scores, non-allergenicity, non-toxicity and the allelic interactions. These epitopes were provided in Table 4.

**Table 4:** The predicted T cytotoxic cells epitopes, their antigenicity scores from the polymerase, envelope, and transacting factor proteins.

Protein	Epitopes	Alleles	Start	End	PR*	Vaxijen Antigenicity^#^
polymerase protein	GHIEPSLSC	HLA-B*15:09	45	53	0.16	1.9236
		HLA-B*42:01		0.37		
	HIGPRALSK	HLA-A*03:01	585	593	0.23	1.6454
		HLA-B*08:02		0.30		
		HLA-B*27:20		0.86		
	LEARAVAMA	HLA-B*45:01	483	491	0.17	1.5777
	VLDLKDCFF	HLA-A*01:01	105	113	0.90	1.4679
		HLA-A*02:11		0.38		
	AMAAVLHVR	HLA-A*02:50	539	547	0.65	1.3796
	RCEQGAIGV	HLA-C*05:01	326	334	0.27	1.2224
		HLA-A*02:11		0.66		
Envelope protein	RLACWSVKQ	HLA-B*08:03	443	451	0.95	2.3391
	CWSVKQANL	HLA-A*24:03	446	454	0.59	2.1629
	WSVKQANLT	HLA-C*15:02	447	455	0.73	1.7785
	LQNRAAIDF	HLA-A*66:01	474	482	0.73	1.3636
	DFCLSTQSA	HLA-B*14:02	85	93	0.37	1.1516
	AAIDFLLLA	HLA-A*02:06	478	486	0.15	1.1476
	SVKQANLTT	HLA-A*30:01	448	456	0.71	1.0535
	QNRAAIDFL	HLA-B*08:03	475	483	0.34	0.9233
	AVLQNRAAI	HLA-A*32:07	472	480	0.24	0.8515
	YLGKLTMLA	HLA-A*02:03	371	379	0.15	0.8309
	VVTADRHNL	HLA-C*07:01	230	238	0.89	0.7649
Transacting protein	WGVVCILPW	HLA-B*53:01	81	89	0.47	1.5006
	LMWGVVCIL	HLA-B*14:02	79	87	0.85	1.5548
	VIAPQPRPL	HLA-B*15:09	68	76	0.80	1.2564
	IAPQPRPLL	HLA-C*03:03	69	77	0.34	1.2724
	RRKWPHLKP	HLA-B*73:01	46	54	0.22	1.0980
	PQPRPLLLL	HLA-B*40:13	71	79	0.73	0.9919
	AHPAISAEQ	HLA-B*15:09	56	64	0.86	0.9307
	APQPRPLLL	HLA-B*08:02	70	78	0.28	0.8880
	HPAISAEQL	HLA-B*15:09	57	65	0.09	0.8577
	RKWPHLKPL	HLA-B*27:05	47	55	0.54	0.6528
	PLLLLMWGV	HLA-A*02:12	75	83	0.30	0.6014
	QLLAVIAPQ	HLA-A*02:16	64	72	0.69	0.5690
	GTRVWPLGR	HLA-A*31:01	114	122	0.42	0.5094
	ISAEQLLAV	HLA-A*69:01	60	68	0.35	0.4689
	PLAHPAISA	HLA-A*02:50	54	62	0.85	0.4175

*PR: Percentile rank with a score of ≤ 1. ^#^The Vaxijen server for antigenicity threshold was 0.4. All the predicted epitopes were nonallergic and nontoxic.

### Helper T lymphocyte epitopes prediction

The reference sequence of each of the three proteins (polymerase, envelope, and transacting factor) was analyzed against the human alleles (HLA-DR, DQ, DP) using IEDB MHC-1I binding prediction tools with a percentile rank of (≤ 10). A vast amount of epitopes were predicted from the three proteins. The predicted epitopes were analyzed for antigenic, allergenic, and toxic outcomes. A total of 21, 6, and 7 epitopes were obtained from the polymerase, envelope, and transacting factor proteins, respectively. They were elected as T helper cell epitopes due to their high antigenicity scores, non-allergenicity, non-toxicity, and allelic interactions. These epitopes were provided in Table 5.

**Table 5:** The predicted T helper cells epitopes and their antigenicity scores from the polymerase, envelope, and transacting factor proteins.

Protein	Core sequence	Alleles	Start	End	Peptide sequence	*PR	#Vaxijen antigenicity
Polymerase	VLDLKDCFF	HLA-DPA1*01:03/DPB1*04:02	99	116	RGWPLMVLDLKDCFFSIP	0.42	1.4679
	LEPLRLKHP	HLA-DRB1*12:01/HLA-DRB1*11:01	161	178	VGQVLEPLRLKHPSLRML	2.60	1.1931
	PVFVIRKAS	HLA-DQA1*05:01/DQB1*04:02	53	70	CWNTPVFVIRKASGSYRL	2.50	1.0881
	LTLLITKLR	HLA-DRB4*01:03	364	381	VLTLLITKLRASAVRTFG	0.61	1.0175
	LLITKLRAS	HLA-DRB1*08:01	365	382	LTLLITKLRASAVRTFGK	1.30	0.8702
	APVGLVAEP	HLA-DQA1*05:01/DQB1*03:02	230	247	LGSTYVAPVGLVAEPRIA	3.10	0.887
	PLPEGKLVA	HLA-DPA1*01:03/DPB1*04:02	17	34	TPVWIDQWPLPEGKLVAL	2.90	0.8656
	KDCFFSIPL	HLA-DQA1*01:01/DQB1*05:01	102	119	PLMVLDLKDCFFSIPLAE	0.71	0.7918
	ARAVAMALL	HLA-DPA1*01:03/DPB1*03:01	481	498	QQLEARAVAMALLLWPTT	1.20	0.7845
	QQGAPVLSA	HLA-DQA1*01:02/DQB1*06:02	80	97	KLVPFGAVQQGAPVLSAL	2.50	0.776
Envelope	AIDFLLLAH	HLA-DPA1*02:01/DPB1*01:01	470	487	RHAVLQNRAAIDFLLLAH	3.00	1.0368
	TVVTADRHN	HLA-DPA1*01:03/DPB1*04:02	228	245	FTVVTADRHNLFMGSEYC	2.90	1.0245
	QNRAAIDFL	HLA-DQA1*01:02/DQB1*05:02	472	489	AVLQNRAAIDFLLLAHGH	2.90	0.9233
	YLGKLTMLA	HLA-DRB1*10:01	368	385	GPCYLGKLTMLAPKHTDI	1.70	0.8309
	ELQLLGSQS	HLA-DRB4*01:01	165	182	PHELQLLGSQSLPNITNI	3.20	0.6819
	VLQNRAAID	HLA-DRB1*13:02	468	485	SIRHAVLQNRAAIDFLLL	1.90	0.4838
Transacting	WGVVCILPW	HLA-DRB1*04:02	75	92	PLLLLMWGVVCILPWRGW	2.60	1.5006
	VIAPQPRPL	HLA-DRB3*03:01	63	80	EQLLAVIAPQPRPLLLLM	2.70	1.2564
	AHPAISAEQ	HLA-DQA1*05:01/DQB1*03:02	49	66	WPHLKPLAHPAISAEQLL	2.20	0.9307
	RKWPHLKPL	HLA-DPA1*02:01/DPB1*14:01	41	58	MRRWRRRKWPHLKPLAHP	2.10	0.6528
	LLAVIAPQP	HLA-DRB1*10:01	60	77	ISAEQLLAVIAPQPRPLL	2.60	0.5912
	LLLLMWGVV	HLA-DRB1*04:03	76	93	LLLLMWGVVCILPWRGWE	2.10	0.5785
	PLAHPAISA	HLA-DQA1*05:01/DQB1*03:03	49	66	WPHLKPLAHPAISAEQLL	1.90	0.4175

*PR: Percentile rank with a score of ≤ 10. ^#^The Vaxijen server for antigenicity threshold was 0.4. All the predicted epitopes were nonallergic and nontoxic.

### Structure of the assembled vaccine

The entire number of predicted B cell, T cytotoxic, and T helper epitopes from the three proteins of ALV were used in the construction of the vaccine. Adjuvant, linkers, and 6-His-tags were also embedded in the final structure of the vaccine. Thus the final vaccine structure comprised 738 amino acids. The antigenicity score of the assembled vaccine was 0.8535 when examined in the VaxiJen server. Also, the vaccine protein was non-allergic in the AllerTOP server.

### Physiochemical properties of the assembled vaccine

ProtParam server was used to examine the physiochemical properties of the assembled vaccine. The predicted vaccine weighed 77.121 kilo Dalton (kd) and possessed a theoretical isoelectric point of 9.81, indicating the proposed vaccine had an alkaline pH. Negatively and positively charged residues were 33 and 79 respectively. The Extinction coefficient at 280 nm measured in water was shown to be 132,125 assuming all pairs of Cys residues forming cystines. The instability index score (II) was 38.24, indicating a stable vaccine protein, while the aliphatic index score was 78.73, indicating a hydrophilic vaccine. The grand average water affinity was -0.130, suggesting a hydrophilic vaccine.

### Secondary and tertiary structures prediction of the assembled vaccine

**Figure 2:**
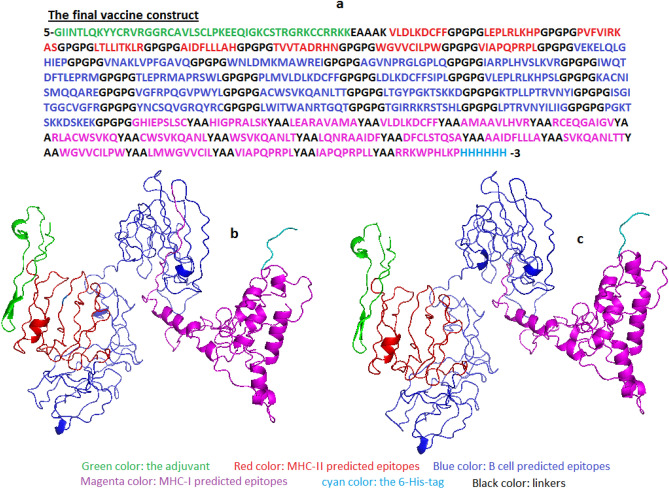
(**a**) The primary sequence of the proposed vaccine. (**b**) The tertiary structure of the vaccine predicted by Raptor X server. **c** The refined structure of the vaccine predicted by the Galaxy web server.

The SS3, ACC, and DISO for the secondary structure were predicted using the Raptor X server. The SS3 showed 23%, 15%, and 61% of the residues as α-helix, β-sheets and coiled, respectively. The ACC provided 49% as exposed residues, 21% as medium residues and 29% as buried residues. The DISO (disordered predicted regions) was 43 (5%). Figure 2 showed the primary sequence, the tertiary and the refined structures of the vaccine construct.

### Vaccine tertiary structure refinement and validation

**Figure 3:**
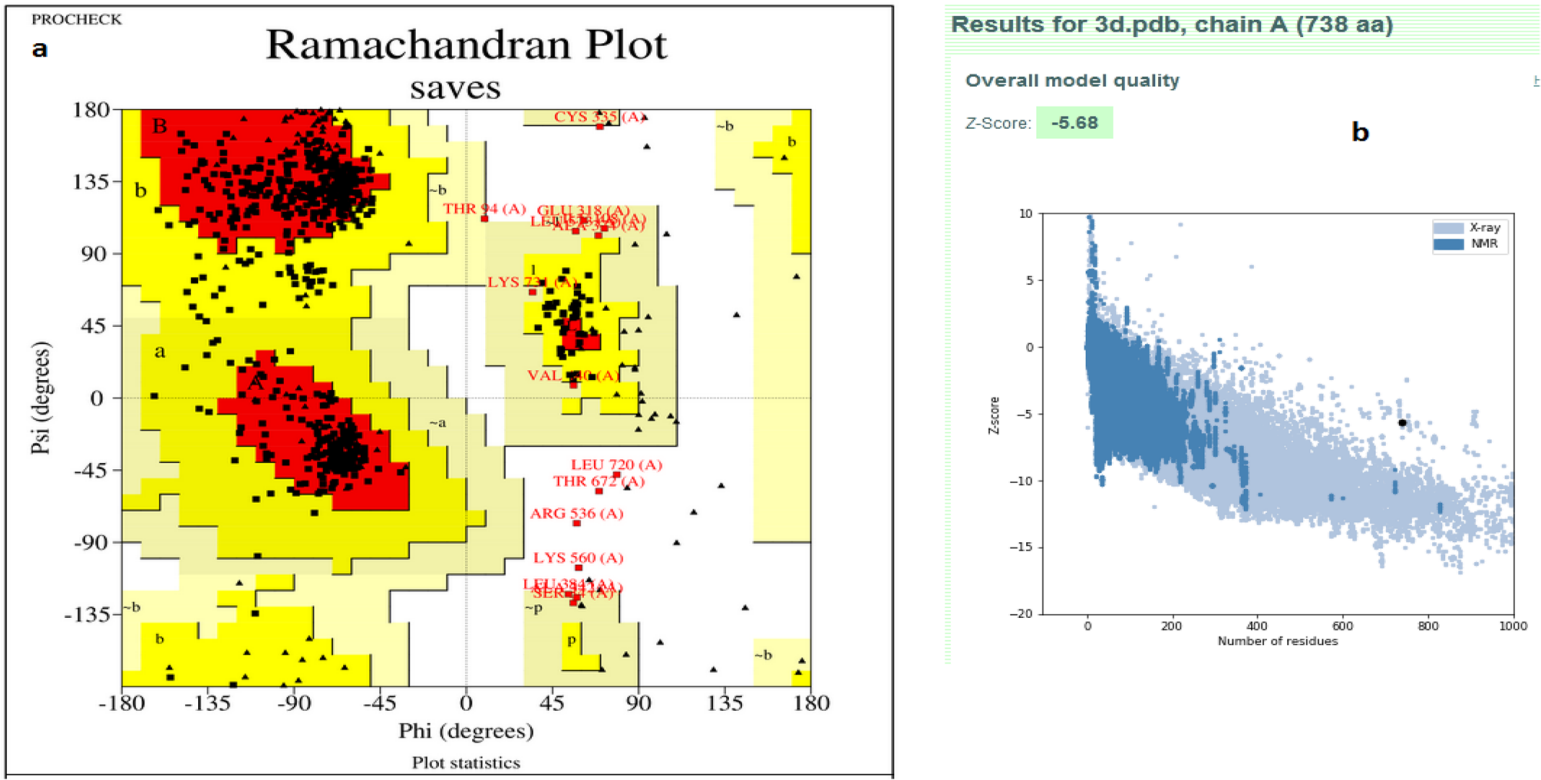
(**a**) In the Ramchandran plot the most favoured region comprised 90.9%; additional allowed region comprised 6.1%, generously allowed region comprised 1.9%, a disallowed region comprised 1.0% of the residues. (**b**) ProSA-server with Z-score of − 5.68.

The vaccine's stability was assessed via the Ramachandran plot after refinementt. In the plot, 90.9% of residues were located in the most favored region. While regions of additional allowed, generously allowed, and disallowed comprised residues of 6.1%, 1.9%, and 1.0%, respectively (Fig. 3a). The ProsA server provided a Z score of -5.68 demonstrating a favorable model structure (Fig. 3b).

### Solubility of the assembled vaccine

**Figure 4:**
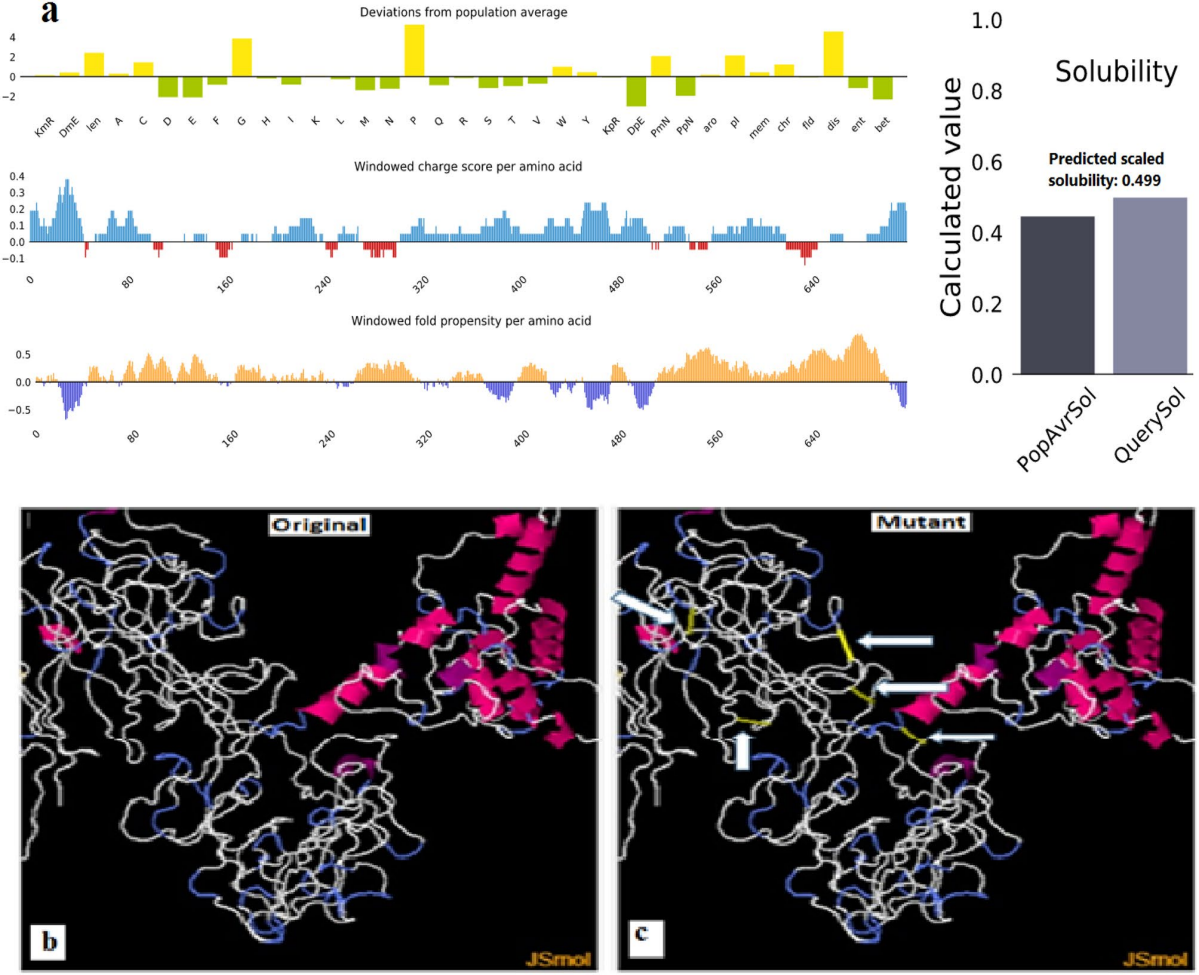
(**a**) The vaccine solubility in comparison to the solubility of *E. coli*. (**b**) Stability of the vaccine protein before disulfide bond engineering in the original form (the form before substitution of amino acids by cysteine). (**c**) The mutant form (the form after substitution of amino acids by cysteine) with five pairs of disulfide bond formation. The disulfide pairs were shown by golden sticks and pointed by white arrows.

Based on the Protein-Sol server, a scaled solubility score of 0.499 was obtained for the vaccine construct, competed with 0.45 for the population solubility of *E*. *coli* (Fig. 4a). As a confirmation, SOLpro was further used to predict the solubility. The probability of the proposed vaccine upon expression on SOLpro was 0.9843, greater than 0.5, provided by the server.

### Stability of the assembled vaccine

By engineering disulfide bonds into the structure of the proposed vaccine, the structural stability of the vaccine was improved. The improvement in stability was made possible by substituting the amino acids in the highly mobile regions in the sequence of the vaccine by cysteine residues. As per the Disulfide by Design 2.0 server, 94 amino acid pairs were identified to form disulfide bonds. However based on the Chi3 angle between + 97 and − 87 and a tolerance of 30 and a maximum Ca–Cb–S angle of 114.60 in the server, five pairs of residues (amino acids) were unstable regions and were replaced by cysteine-cysteine residues. The position and the replaced residues in the vaccine structure were A107-R127; I150-G179; P210-P280; P278-P312 and G500-L538 and were shown in Fig. 4b,c.

### Immune simulation

**Figure 5:**
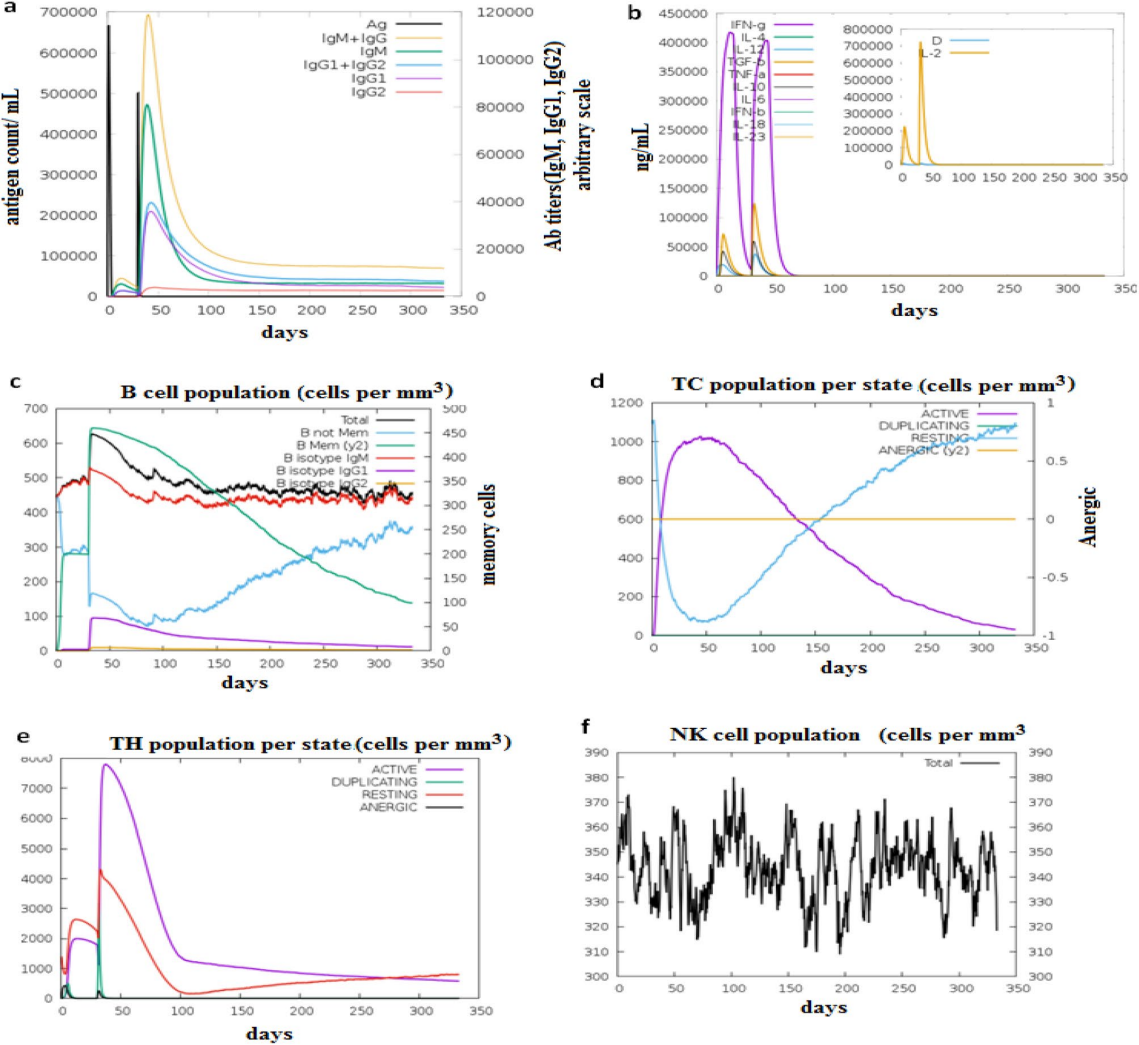
The immune simulation of the predicted vaccine after the two injections of the antigen. (**a**) Antibodies production in response to antigen injections (antibodies were shown as different colored peaks and the antigen was shown in black color). (**b**) The induced cytokines secretion and the IL-2 level with the measure of diversity. (**c**) Showed the memory, not memory and the isotypes of B-cell populations. (**d**) Showed the active T-cytotoxic (TC) cell populations. (**e**) Showed the active T-helper (TH) cell populations. In (**d**, **e**) The resting state demonstrated the cells not provided with the antigen (vaccine). The anergic state demonstrated tolerance of the T-cells to the antigen due to repeated exposures. (**f**) Natural killer cell populations.

The obtained immune simulation results were coincided with actual immune responses. This was proved by marked increase in the primary, secondary and tertiary immune responses accompanied by drop in the antigen concentration (Fig. 5a). The cytokines and interleukins (IL) levels during the injections showed that the IL-2 level was compatible with the measure of diversity (Simpson index, D) (Fig. 5b). The elevation of the measure of diversity over time is considered as danger signal together with leukocyte growth factor. Therefore, the lower the measure of diversity value, the lower the diversity. In addition the primary response, for instance, was featured by augmented IgM level, while, secondary and tertiary responses provided marked elevation in the population of B-cells and the antibodies level (Fig. 5c). This showed the development of immune memory accompanied by rapid clearance of the antigen upon subsequent exposures. Moreover the population of T-cytotoxic (TC) (Fig. 5d) and T-helper (TH) (Fig. 5e) lymphocytes showed high response level coincided with memory development. The natural killer cells maintained high levels throughout the duration of exposure (Fig. 5f).

### Molecular dynamic simulation (MD)

**Figure 6:**
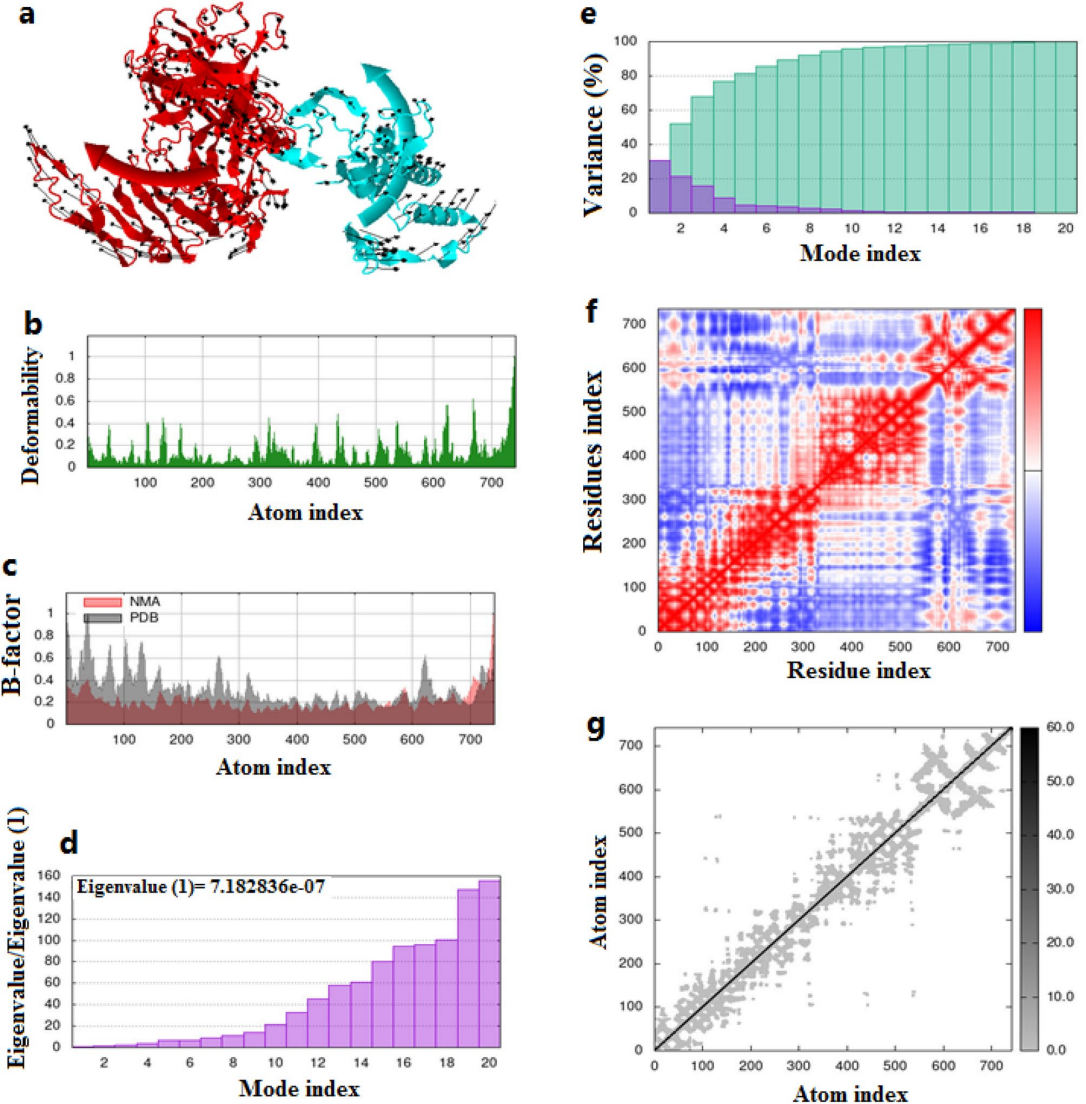
Showed the MD of the vaccine protein complex. (**a**) The direction of the motion was shown by the red and cyan colors. (**b**) The stability of the vaccine was analyzed by the low main chain deformability. (**c**) The B factor/ mobility. (**d**) The Eigenvalue demonstrated the protein’s normal mode and the stiffness of the motion. (**e**) The normal mode variance and (**f**) is the covariance matrix. (**g**) the elastic network model showed a stiffer mode of the residues.

A Normal mode analysis (NMA) was performed on the MD of the vaccine protein using the iMODS server. As shown in Fig. 6a, the arrows indicated the direction in which each vaccine protein residue moves. Deformability was also demonstrated with hinges in the chief chain, as a result of an individual distortion of the residues (Fig. 6b). Experimental B-factors were calculated on the basis of the PDB field and the NMA data (Fig. 6c). A normal mode of deformability of the vaccine structure was shown by the eigenvalue, which directly correlated to the energy required with the deformability. The obtained eigenvalue (7.182836e-07) demonstrated the stiffness of the motion (Fig. 6d). The lower eigenvalue is always associated with the easier deformation of the protein structure. The normal mode variance is inversely related to the eigenvalue. Figure 6e illustrated the cumulative variance and individual variance as green and purple bars, respectively. It was possible to determine the correlations between proteins by examining the covariance matrix (Fig. 6f). Thus, red identified correlated motions, white indicated uncorrelated motions, and blue indicated anti-correlated motions. Spring-connected or joined atom pairs were demonstrated in the elastic network model. A single-atom pair spring was represented as a dot, and colored according to its stiffness, with darker dots denoting stiffer strings, and vice versa (Fig. 6g).

### Discontinuous B-cell epitopes prediction

**Figure 7:**
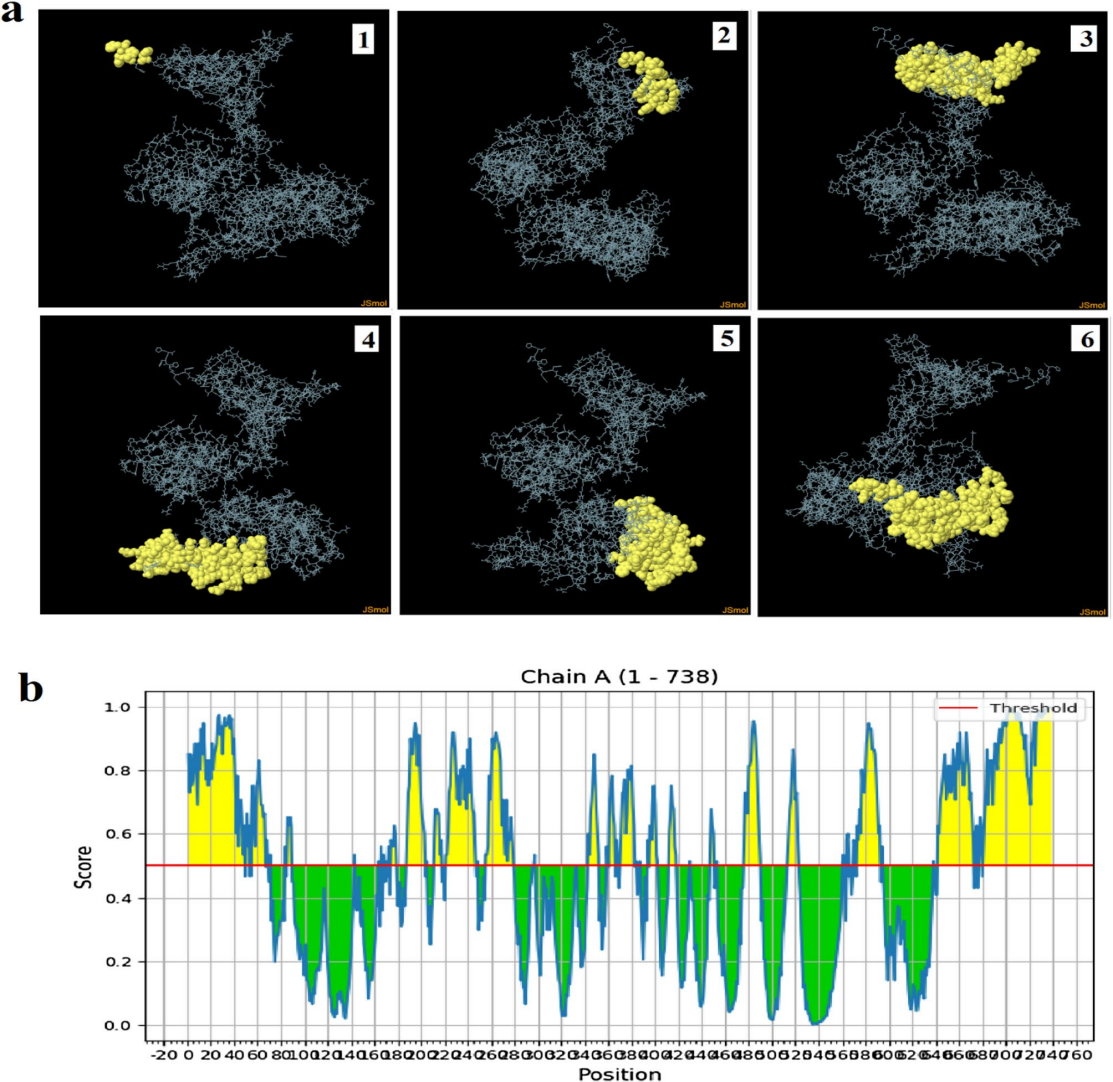
(**a**) showed the 3D structures of six discontinuous B-cell epitopes predicted by the ElliPro (1–6). Epitopes were shown in yellow color, while grey color showed the constructed vaccine. (**b**) The yellow color demonstrated the discontinuous epitopes while the green color was the continuous epitopes. The red line showed the threshold of the residues score.

Table 6 and Fig. 7 demonstrated six discontinuous B cell epitopes. The scores of these epitopes were ranged from 0.996 to 0.615 with a total of 405 predicted residues. The size of the conformational epitopes ranged from 4 to 108 residues.

**Table 6:** The number of the predicted discontinuous B cell epitopes with the number of the residues and their scores.

No	Residues	Number of residues	Score
1	A:H734, A:H736, A:H737, A:H738	4	0.996
2	A:R717, A:P718, A:L719, A:L720, A:Y721, A:A722, A:A723, A:R724,	16	0.891
3	A:K560, A:D561, A:F564, A:Y565, A:A566, A:A570, A:A571, A:L573, A:H574, A:V575, A:R576, A:Y577, A:A578, A:A579, A:R580, A:C581, A:E582, A:Q583, A:G584, A:A585, A:I586, A:G587, A:V588, A:Y589, A:A590, A:A591, A:R592, A:L593, A:A594, A:C595, A:C642, A:L643, A:S644, A:T645, A:Q646, A:S647, A:A648, A:Y649, A:A650, A:A651, A:A652, A:A653, A:I654, A:D655, A:F656, A:L657, A:L658, A:L659, A:A660, A:Y661, A:A662, A:A663, A:S664, A:V665, A:K666, A:Q667, A:A668, A:N669, A:L670, A:T671, A:T672, A:A674, A:A675, A:G677, A:V678, A:V679, A:I681, A:L682, A:P683, A:W684, A:Y685, A:A686, A:A687, A:L688, A:M689, A:W690, A:G691, A:V692, A:V693, A:C694, A:I695, A:L696, A:Y697, A:A698, A:A699, A:V700, A:I701, A:A702, A:P703, A:Q704, A:P705, A:R706, A:P707, A:L708, A:Y709, A:A710, A:A711, A:I712, A:A713, A:P714, A:Q715, A:P716	102	0.78
4	A:G1, A:I2, A:I3, A:N4, A:T5, A:L6, A:Q7, A:K8, A:Y9, A:C11, A:R12, A:V13, A:R14, A:G15, A:G16, A:R17, A:C18, A:A19, A:V20, A:L21, A:S22, A:C23, A:L24, A:P25, A:E28, A:Q29, A:I30, A:G31, A:K32, A:C33, A:S34, A:T35, A:R36, A:G37, A:R38, A:K39, A:C40, A:C41, A:R43, A:E46, A:A47, A:A48, A:A49, A:K50, A:V51, A:L52, A:D53, A:K55, A:D56, A:C57, A:F58, A:F59, A:G60, A:P61, A:G62, A:P63, A:G64, A:L65, A:E66, A:P67, A:L68, A:L70, A:K71, A:H72, A:V82, A:R84, A:K85, A:A86, A:S87, A:G88, A:P89, A:G90, A:P91	73	0.738
5	A:W143, A:G144, A:L167, A:Q168, A:L169, A:G170, A:H171, A:I172, A:E173, A:P174, A:G175, A:P176, A:G177, A:P178, A:G179, A:N181, A:A182, A:G188, A:A189, A:V190, A:Q191, A:G192, A:P193, A:G194, A:P195, A:G196, A:W197, A:N198, A:L199, A:D200, A:M201, A:K202, A:M203, A:A204, A:W205, A:E207, A:P210, A:G211, A:P212, A:G213, A:A214, A:G215, A:V216, A:N217, A:P218, A:R219, A:G220, A:L221, A:G222, A:P223, A:L224, A:Q225, A:G226, A:P227, A:G228, A:P229, A:G230, A:I231, A:A232, A:R233, A:P234, A:L235, A:H236, A:V237, A:S238, A:L239, A:K240, A:V241, A:R242, A:G243, A:P244, A:G245, A:P246, A:G247, A:I248, A:Q250, A:F253, A:T254, A:L255, A:E256, A:P257, A:R258, A:M259, A:G260, A:P261, A:G262, A:P263, A:G264, A:T265, A:L266, A:E267, A:P268, A:R269, A:M270, A:A271, A:P272, A:R273, A:S274, A:W275, A:L276, A:G277, A:P278, A:G279, A:P280, A:G281, A:P309, A:P312, A:G313	108	0.647
6	A:A342, A:E344, A:G345, A:P346, A:G347, A:P348, A:G349, A:V350, A:G351, A:F352, A:G356, A:P358, A:W359, A:Y360, A:L361, A:G362, A:P363, A:G364, A:P365, A:G366, A:A367, A:C368, A:W369, A:S370, A:V371, A:K372, A:Q373, A:A374, A:N375, A:L376, A:T377, A:T378, A:G379, A:P380, A:G381, A:P382, A:G383, A:L384, A:T385, A:G386, A:Y387, A:P388, A:K394, A:D395, A:G396, A:P397, A:G398, A:P399, A:G400, A:K401, A:T402, A:P403, A:V409, A:N410, A:Y411, A:I412, A:G413, A:P414, A:G415, A:P416, A:G417, A:I418, A:S419, A:R429, A:G430, A:P431, A:R445, A:C446, A:G447, A:P448, A:G449, A:P450, A:G451, A:L452, A:W453, A:R475, A:S476, A:T477, A:S478, A:H479, A:L480, A:G481, A:P482, A:G483, A:P484, A:G485, A:L486, A:P487, A:T488, A:R489, A:V490, A:N491, A:Y492, A:K514, A:G515, A:P516, A:G517, A:P518, A:G519, A:G520, A:H521, A:I522	102	0.615

### Active site detection in the vaccine structure

**Figure 8:**
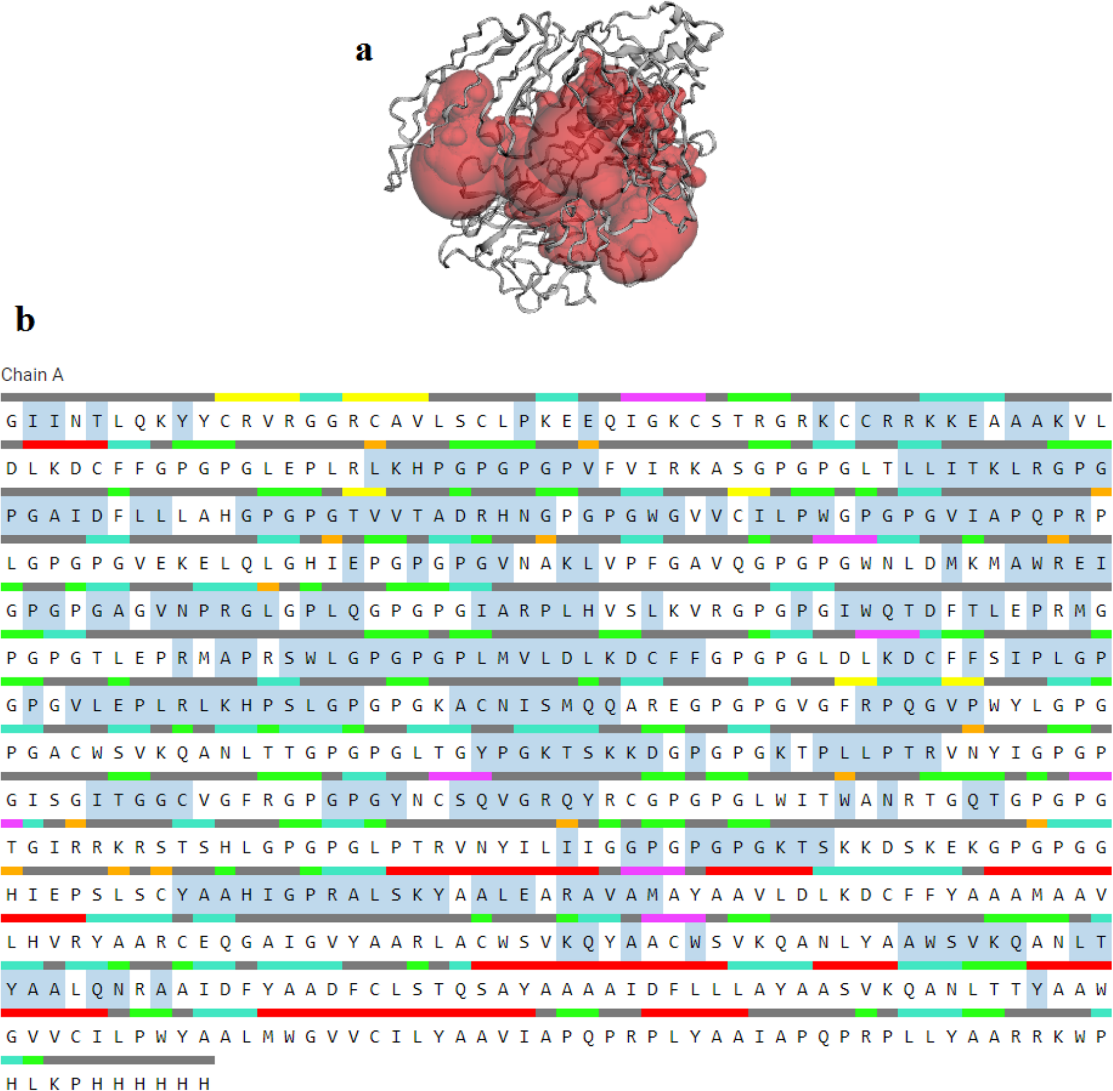
(**a**) The pocket panel (shown in red color) in the structure of the vaccine. (**b**) the sequence and annotation panels in the vaccine construct.

Prior to docking the active binding pocket of the vaccine construct was predicted by CASTp. The highest pocket area and volume were 7086.109 Å2 and 18,104.055 Å3, respectively. This result indicated pocket area and volume were suitable for binding during the molecular docking. The surface binding pocket of the vaccine and the sequence were shown in Fig. 8.

### Molecular docking of the vaccine protein with chicken TLR7

**Figure 9:**
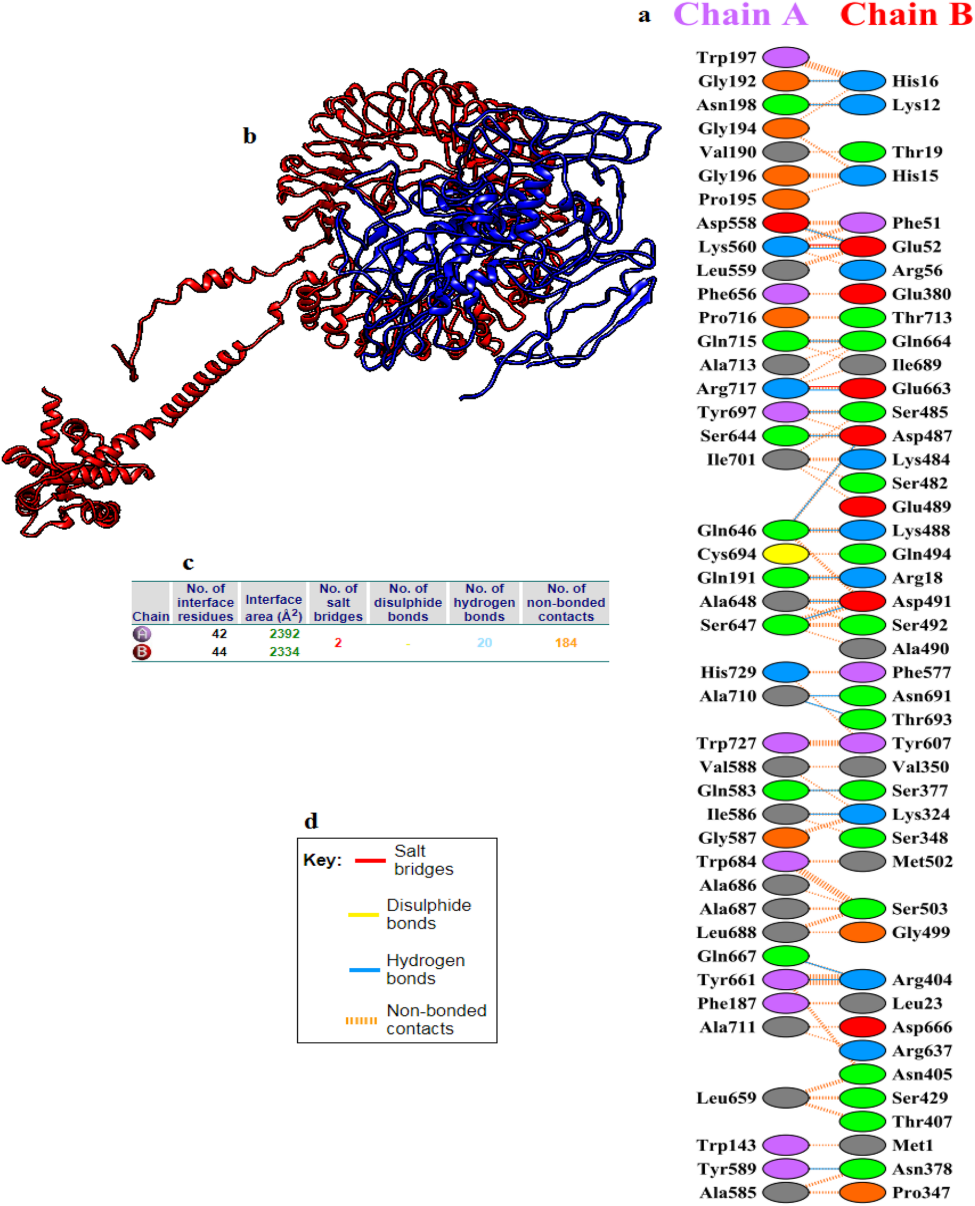
Molecular docking interaction between the vaccine construct with chicken TLR7. (**a**) Interacting residues between the vaccine (chain A) and TLR7 (chain B). (**b**) Chicken TLR7 (red color) and the vaccine construct (blue color) docked complex. (**c**) interface statistics result. (**d**) Key showing the residue interactions across interface between the docked molecules.

The interaction between the vaccine construct and chicken TLR7 was assessed by HADDOCK software. HADDOCK clustered 13 structures in 3 cluster(s), which represents 6% of the water-refined models. Upon refinement, 20 structures were grouped into one cluster, resulting in 100% of the HADDOCK water-refined version. The binding affinity between the vaccine and the chicken TLR7 was − 263.0 ± 3.1 demonstrating the strong binding between the molecules. As shown in Fig. 9, this binding was evident by 20 hydrogen bonds, 2 salt bridge, and 184 non-bonded contacts. These bonding events between the amino acids of the molecules were provided in Table 7. Additionally PRODIGY web server showed binding affinity in terms of Gibbs free energy (ΔG) and thermodynamics (dissociation constant) between the docked molecules. Such kind of binding affinity decided the real interaction between the docked molecules under certain circumstances within the cell. The server showed ΔG values − 21.1 kcal/mol for the vaccine construct and chicken TLR7 and the dissociation constant was 3.1e−16 indicating the docked molecules were energetically viable.

**Table 7:** List of Atom − Atom Interactions between the Vaccine and chicken TLR7 Interface.

			Vaccine			Hydrogen bonds			Chicken TLR 7			
	Atom number	Atom name	Residue name	Residue number	Chain	< – >	Atom number	Atom name	Residue name	Residue number	Chain	Distance
1	1687	OE1	GLN	191	A	< – >	6785	NH1	ARG	18	B	2.73
2	1697	O	GLY	192	A	< – >	6766	NE2	HIS	16	B	3.2
3	1743	OD1	ASN	198	A	< – >	6723	NZ	LYS	12	B	2.7
4	4893	OD2	ASP	558	A	< – >	7105	N	GLU	52	B	2.71
5	4912	NZ	LYS	560	A	< – >	7112	OE2	GLU	52	B	2.59
6	5133	OE1	GLN	583	A	< – >	10,298	OG	SER	377	B	3
7	5158	O	ILE	586	A	< – >	9786	NZ	LYS	325	B	2.76
8	5182	OH	TYR	589	A	< – >	10,308	ND2	ASN	378	B	3.05
9	5715	OG	SER	644	A	< – >	11,439	OD1	ASP	487	B	2.62
10	5734	OE1	GLN	646	A	< – >	11,450	NZ	LYS	488	B	2.69
11	5735	NE2	GLN	646	A	< – >	11,440	OD2	ASP	487	B	3.02
12	5740	N	SER	647	A	< – >	11,477	OD1	ASP	491	B	2.85
13	5748	N	ALA	648	A	< – >	11,477	OD1	ASP	491	B	2.7
14	5865	OH	TYR	661	A	< – >	10,580	NH1	ARG	404	B	2.98
15	5916	OE1	GLN	667	A	< – >	10,580	NH1	ARG	404	B	2.79
16	6195	OH	TYR	697	A	< – >	11,417	OG	SER	485	B	2.72
17	6312	O	ALA	710	A	< – >	13,440	ND2	ASN	691	B	3
18	6312	O	ALA	710	A	< – >	13,458	OG1	THR	693	B	3.11
19	6348	NE2	GLN	715	A	< – >	13,192	OE1	GLN	664	B	2.83
20	6369	NH1	ARG	717	A	< – >	13,182	OE21	GLU	663	B	3.01

			Vaccine			Salt bridges			Chicken TLR 7			
1	4912	NZ	LYS	560	A	< – >	7111	OE1	GLU	52	B	2.59
2	6369	NH1	ARG	717	A	< – >	13,183	OE2	GLU	663	B	3.01

### In silico molecular cloning

**Figure 10:**
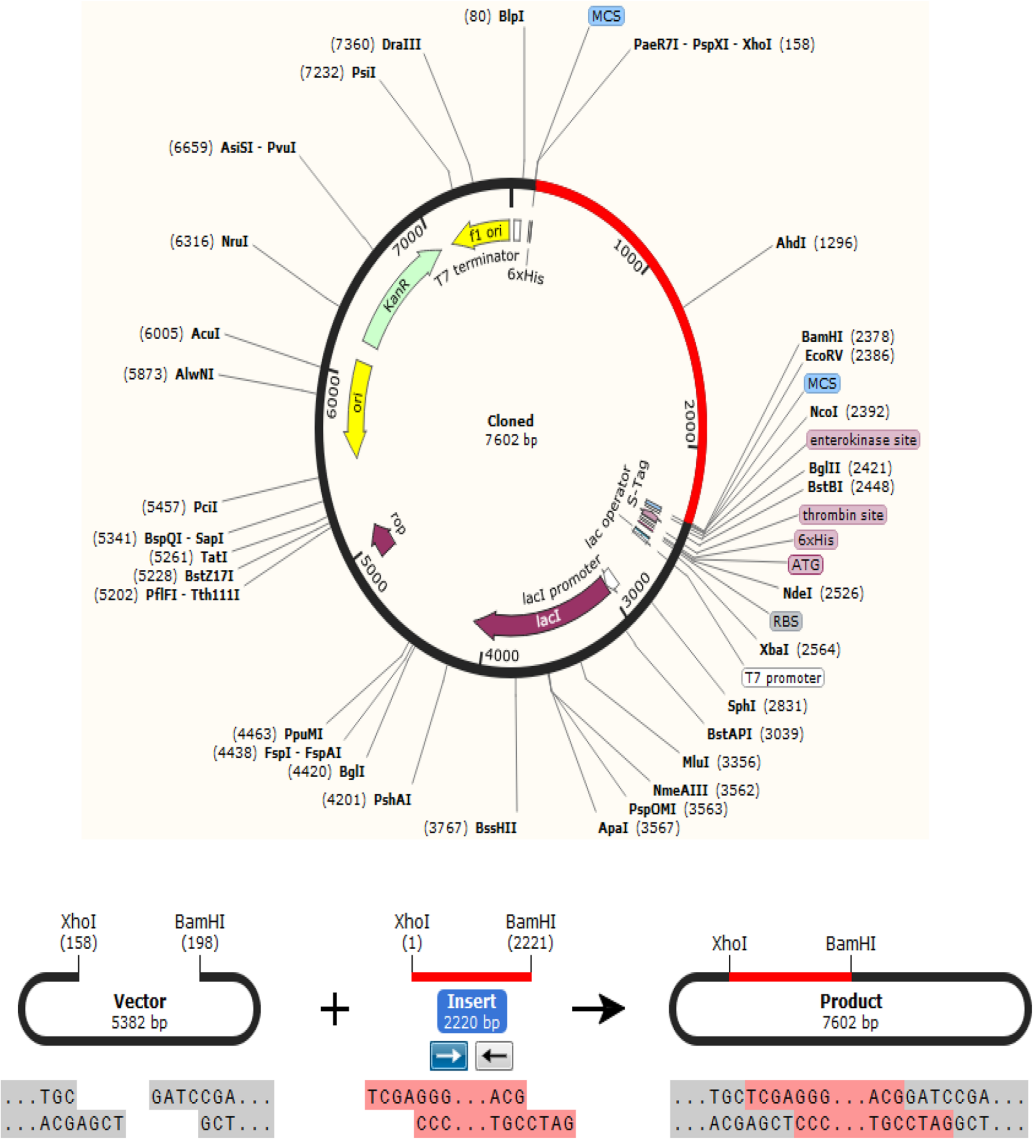
The vaccine DNA sequence was cloned in the pET30a ( +) vector. The vector was shown in black colour, while the red colour represents the gene coding for the vaccine protein.

The potential host expression of the target protein was performed by JCAT. The protein sequence of the vaccine was reversibly translated into DNA sequence. The index codon adaptation value of the DNA sequence was equal to 1.0, indicating a higher proportional abundance of the common codons. The improved GC content value of the DNA sequence was 59.53%, providing significant GC content. As shown in Fig. 10 the DNA sequence was cloned into the pET-30a(+) vector utilizing Snapgene software between the restriction enzyme cutting locations of BamH1 and Xho1.

## Discussion

The most common avian retrovirus that causes a variety of neoplastic diseases in chicken is the avian leukosis virus (ALV)^2^. Globally, the ALV morbidity and mortality rates contributed to the poultry industry’s economic decline^1,3^. This is accompanied by subsequent adverse effects on the food supply worldwide. Preventing and controlling viral infection in avian industry is always via mass vaccination means. Therefore vaccines designed to combat avian viral diseases will significantly alleviate selection pressure on the virus and on the field strains^68^. Concerning ALV infection in poultry, many anti-ALV vaccines were developed, but they targeted only specific strains. Also some of the vaccine trials had less immunogenicity and limited protection^69^. Currently, neither known treatment nor vaccination against ALV is available. Multiple studies used the multi-epitope vaccine prediction against ALV and evaluated their possibility as effective vaccine candidate via challenging in chickens^70–72^. For instance, one study provided a novel oral vaccine of recombinant gp85 protein in *L. plantarum* with a significant increase in antibodies post-inoculation^72^. The study demonstrated a protection against ALV-J and showed protective immune response against early ALV-J infection based on viremia analysis^73^. Another study showed the impact of polysaccharides from *Ulvapertusa* as anti-ALV-J. The polysaccharides demonstrated strongest suppression of the ALV-J activity as they bound with the viral particles and obstacle ALV-J adsorption by host cells accompanied by significant reduction of gp85 protein expression^74^. However these studies reported partial immune protections against ALV-J infections in chickens.

In this study a vaccine with multi-epitopes was designed and showed increased immunogenicity and enhanced immune responses as a result of the existence of epitopes from various target genes. Also the designed vaccine activated the humoral and cell-mediated immunity as previously described^75^. These could solve limitations occurred during controlling the ALV infection^75,76^. Most importantly, the safety and effectiveness, allergenicity and the immunogenicity of the predicted vaccine were also taken into consideration to ensure the safety of the designed epitopes^77^. In addition, the toxic effect, the solvent accessibility of the amino acids, the identification of B cells, and MHCmolecules were also contemplated to ensure the effectiveness of the predicted epitope vaccine. All these measures give the predicted vaccine an advantage over the traditional ones for controlling the ALV infection.

Thus the conserved predicted epitopes from ALV proteins were submitted to the ABCpred server. Based on ANN, Hidden Markov model (HMM) and support vector machine (SVM) in the ABCpred server the B cell epitopes were predicted^78^. Furthermore, the predicted epitopes were subjected to antigenic, allergenic and toxic analysis to confirm their suitability as B cell epitopes. Also T cell epitopes were predicted from their reference sequences using the IEDB server. In addition to their high binding affinity to MHC alleles, the predicted epitopes demonstrated high antigenicity score in VaxiJen server, and they revealed no allergic or toxic characteristics. Therefore they were picked to enter the vaccine protein structure. With the aid of expedient linker sequences (protein spacers), the generated B- and T-cell epitopes were fused together^49,52^. Linkers are crucial to the assembly of stable, bioactive fusion proteins. Essentially, linkers reduced the likelihood of junctional antigen formation as well as enhancing antigen processing and presentation^52^. They are also important to construct and facilitate structural flexibility and reduced rigidity^52^. A sequence with the least junctional immunogenicity was generated in this study using the linkers GPGPG and YAA. The GPGPG linkers were applied to facilitate immune processing and merge the B-cells and T-helper cell epitopes. The YAA linkers ameliorated the immunogenicity of a vaccine by impacting protein stability and epitope presentation capacity and were used to fuse the cytotoxic T-cells^24,79^. As an adjuvant, the β-defensin was added via an EAAAK linker at the N terminus of the vaccine construct to improve the immunogenicity of the vaccine. EAAAK are helical linkers used to control the distance and decrease the interference between the domains^24,79^. As a 45amino acids peptide with a relatively small size, the β-defensin was used for its immune modulation and antimicrobial features^44^. To facilitate purification and downstream testing, a small 6His tag was added to the proposed vaccine at the C-terminal to prevent protein structure from being altered^80^.

The stability of the vaccine was confirmed by the ProtParam server based on its physiochemical properties. VaxiJen and AllerTOP servers were used to assess the antigenic and allergenic features of the vaccine. The results indicated that the vaccine was antigenic without causing any allergic reactions. In order to select the best score of the model generated by the 3D structure of the vaccine protein, the secondary and tertiary structures of the vaccine construct were analyzed. The Ramachandran plot showed favorable results in the distribution of the vaccine residues and provided a stable structure. The ProSA server indicating that the overall model is suitable for acceptance as a potential ALV vaccine^51,52^.

The solubility of the designed vaccine in this study was calculated with the protein-sol and SOLpro servers. As a comparison with the solubility of *E coli*, Protein-sol presented the vaccine as a soluble protein and predicted a scaled solubility of 0.499, an increase over 0.45 from the average solubility of the *E. coli* population. According to the SOLpro server, the predicted solubility was 0.9843, which confirmed this result. To obtain disulfide bonds between the vaccine residues, the proximity and geometry composition of the residue pairs were evaluated for the formation of disulfide bonds. Five unstable regions in the vaccine structure were replaced by the formation of disulfide bonds. Disulfide bonding increases the stability of the vaccine protein as previously stated^51,52^.

Immune simulation demonstrated results that consistent with the real immune responses. Generally there were elevated levels of the immune responses after repeated exposure to the vaccine (antigen). In addition, there was marked development in the memory cells of B and T lymphocytes. Most importantly, IL-2 and IFN-γ were elevated following the initial injection and provided peak levels after antigen repeated exposures, showing the high levels of T-helper lymphocytes and efficient immunoglobulin production. The Simpson index demonstrated a possible different immune response, indicating the vaccine structure contains multiple B and T cells epitopes^44^. A study by Landman et al., demonstrated the interaction of the NK cells during ALV infection^81^. They showed that during ALV infection in immunosuppressed chicken, the NK cells provided reduced killing activity than the NK cells of the uninfected controls. Natural killer cells play a paramount defense mechanism in host and surveillance of tumor, resulting in cell death and secretion of cytokines and chemokine. Moreover, NK cells have a significant role in immune regulation of T cells and DC functions during viral infection in mouse models^81^. In addition to that, there is scarcity in ALV vaccine researches concerning the immune system of chickens. Thus vaccine research studies may provide a realistic importance and increase the knowledge of immune system of ALV with great insights into human retroviral diseases^69^.

Molecular dynamics simulation (SD) was used to assess the complex stability of the vaccine protein. In previous studies, macromolecule stability was associated and correlated with the fluctuations of atoms^82,83^. Therefore MD was performed to evaluate the essential dynamics and complex stability of the vaccine based on the protein normal modes in the iMODS server. The analysis showed that no atoms had a significant distortion in the vaccine protein structure indicating less chance of deformability with proper stiffness motion.

It is noteworthy that bioinformatics and immunologic analysis tools provided that the chimeric vaccine should comprises linear and discontinuous B-cell epitopes in addition to MHC-I and MHC-II epitopes^84^. Our predicted vaccine was shown comprising all these epitopes which strongly facilitate the interaction against the humoral and adaptive immunity of the host^84^.

The geometry and topology features of protein structures, such as interior cavities, pockets in the structure surface and the cross channels prior to the docking process are essential to study the function of proteins. The vaccine construct showed surface binding pocket suitable for docking with chicken TLR7. Based on the molecular docking, the constructed vaccine and the TLR7 demonstrated a good binding affinity. The vaccine strongly bound to the chicken TLR7 revealed by the negative values of the docking process^62^. Among the ten chicken TLRs, TLR7 has a propensity to recognize the viral constituents located on the extracellular surfaces^63^, thus has the advantages to be elected for docking against ALV predicted vaccine.

Molecular cloning is an important step to produce recombinant vaccines. Prior to cloning into the pET-30a [+] vector, reverse transcription and adaptation of the vaccine protein to DNA by the JCAT were performed on *E. coli* strain K12. The DNA sequence showed a Cal-Value of 1.00 and a GC ratio of 59.57%, demonstrating a high expression in bacteria. Cloning of the vaccine construct gene in the vector was typically carried out in multiple cloning sites. This result provided prolific cloning of the vaccine protein.

## Conclusion

This study demonstrated the urgency need for effective vaccine strategy against ALV due to the lack of treatment or approved antiviral drugs. This study inclusively exploited the computational and immunoinformatics approaches to design and evaluate a multi-epitope vaccine candidate against ALV. Constructing abjunctive vaccine with antigenic characteristics, devoid of allergenicity and toxicity is a crucial footstep to combat ALV. This study provided a potential vaccine epitopes with immunogenic adjuvant and suitable linkers. The vaccine was stable and provokes strong immune response interactions. Moreover the vaccine showed favorable interaction with the chicken immune receptor as confirmed by molecular docking analysis. However, validation of this vaccine via experimental studies is essential to guarantee the immunogenicity and protective efficacy of the vaccine.

## Data Availability

The datasets generated during and/or analyzed during the current study are available from the corresponding author on reasonable request.
